# Case of Malignant Tumour of the Os Uteri, Excised during Labour

**Published:** 1848-09

**Authors:** J. M. Arnott

**Affiliations:** Surgeon to the Middlesex Hospital, and President of the Society


ROYAL MEDICAL AND CHIRURGICAL SOCIETY.

*Case of Malignant Tumour of the Os Uteri, excised during Labour.* By J. M. Arnott, Surgeon to the Middlesex Hospital, and President of the Society.—The author first referred to cases of scirrhus, cancer, and other malignant growths of the uterus, in a state of pregnancy, proving the frequent fatality of this complication. He also adverted to the opinion maintained by some authors regarding the impropriety of excising the diseased parts under such circumstances, and considered the case he was about to narrate as illustrative of both the advisability and safety of the step in question. The subject of the disease (a lady aged thirty eight) applied to the author, in 1844, for an indurated enlargement of the anterior lip of the os uteri, which was rugged and granular near to the os itself; this was not tender, but bled under the examination. A consultation with Drs. Ferguson and Locock being held, it was agreed that the disease admitted of removal, as the neighbouring portion of the os uteri was healthy; but that, as the patient was at the time in the fifth month of pregnancy, it was desirable to defer operating until after the patient's confinement. Four months afterwards, the author was summoned to meet Dr. Locock, in consequence of labour being protracted by the presence of the morbid growth. This was still limited to the anterior lip and right side of the uterus, and had attained the size of a large green walnut: the tissue beyond and around appeared healthy. The diseased mass and contracted os uteri were forced down almost into view when a pain came on: hooks were fixed to the former, which was excised by a succession of strokes with curved scissors, and scarcely any blood was lost. Immediately afterwards, the os uteri expanded uniformly, and in a quarter of an hour a healthy child was born. The patient had a good recovery. On section the tumour presented a yellow colour and fibrous appearance. After an interval of some months, this patient was again the subject of frequent hæmorrhage; and it was then discovered that the posterior lip and cervix uteri were the seat of a similar disease, which extended this time beyond reach. She lived until June, 1846, sixteen months after her confinement, when she died with all the symptoms of malignant disease of the womb. The author remarked that, in this instance, the lives of both mother and child were saved by the timely surgical interference; but he did not regard the case as illustrative of the usual condition met with in similar cases, which are rarely of a nature to admit of an operation under such favourable circumstances. He then narrated a case which occurred under his notice in the Middlesex Hospital, and in which the cancerous disease was much more extensive. After protracted labour, the os uteri was rent during a violent pain, and before the author arrived to relieve the tension by incisions. Two fœtuses were born at the sixth month, and the patient subsequently rallied. The author concludes by remarking, that in cases similar to the last alluded to, incisions offer the first and best means of relief; but that when the malignant growth is confined to a limited portion of the os uteri, the alternative of excision, as in the case first related, should be entertained.

Dr. Locock would make a small addition to the first case detailed in the paper. Four or five weeks after delivery, the patient seemed quite well; there was no portion of disease to be detected; and only a small cicatrix left from the operation, a cicatrix which was scarcely to be felt. This lady, having some anxious feelings respecting her case, kept the early symptoms of its return to herself, and it was only after repeated hæmorrhages, and at the expiration of nine months, that he (Dr. Locock) had seen her. The disease had then spread to the opposite side of the uterus, and pressed upon the rectum. The period was then gone by for any remedial means to be employed. The question of the propriety of operating in cases of this kind, during labour, had been mooted. This case showed that such operation was desirable. In those cases, where it was impossible to bring away a living child without operation on the tumour, he could not think that we were justified in sacrificing the lives of both mother and child by non-interference—a result which must occur if we did not use means to prevent it. It was, at all events, the salvation of one life out of two. He should never hesitate, under such circumstances, to attempt to save the life of the child, when possible, without exposing the mother to more risk than she would experience by not operating. Now, in such a case as this, what was the practitioner to do? It was quite clear that there was only one proceeding which offered the least chance to the child—that proceeding was an operation. And let it be borne in mind, that if the child were not attempted to be saved in this manner, there was as much risk to the life of the mother by any other proceeding. Incising the uterus, no doubt, must be done at the risk of the mother, but, if successful, might delay the progress of the disease for some time, though occasionally, as was known, the disease spread more rapidly after operative means had been used for its removal. Still we must never forget, in these cases, that without operation, both lives would be destroyed, with it, probably only one, and perhaps neither.

Mr. Lloyd related a case of cancer of the uterus, affecting the anterior lip of the organ, which was tumefied and thickened. He could pass his finger quite beyond the diseased portion. Various applications were made to this growth, without any beneficial result, and the pain and the discharge became so great that the life of the patient was threatened. Under these circumstances it was determined to remove the diseased portion, and as he was fearful of at once excising so large a part of the uterus, he passed a ligature entirely round the cervix uteri, by which he completely encompassed the tumour. There was some hæmorrhage, and a considerable discharge of serous fluid, tinged with blood; the whole of the enclosed portion swelled enormously, so that the os uteri presented itself at the external orifice. There was so much pain that it was considered desirable to remove the diseased portion at once; this was accordingly done by a stroke of the knife, and the patient made a good recovery. A fungus, however, presented itself at the point of operation, to which escharotics were applied with complete success. The patient remained well for two years, when ulceration set in and eventually destroyed the greater portion of the uterus. The patient sank, and on examination after death, there was no trace of cancer in any other structure of the body. In three other cases he had removed portions of the os uteri for fungoid disease. In two of these cases the removal was effected partially by ligature and partly by caustic, and finished by an elliptical incision with the scissors. He eulogized the use of the potassa fusa and nitrate of silver in these cases; they prevented hæmorrhage, and sometimes cured the disease.

Dr. Locock said, that in excising portions of the os uteri for diseased growths, it was not necessary to use a ligature; there would be no alarming hæmorrhage from excision. In his opinion, the ligature did more harm than good. He (Dr. Locock) applied caustics after, rather than before, the operation, with the view of getting rid of any portion of the disease which might be left by the knife, and of arresting hæmorrhage.

Mr. Arnott said, that the question of the propriety of removing tumours from the os uteri was one of great importance. In some cases the tumours might be entirely removed, but in other instances you could excise them only. In the first case detailed, excision was resorted to, and there was no hæmorrhage; in the second case, the disease was beyond reach, and the parts were incised only. In the case described in the paper by Dr. West, he (Mr. Arnott) had been called to the patient at twelve at night; the os uteri was situated high up, and there was great difficulty in reaching it to incise it. Notwithstanding what had fallen from Dr. Locock, you might have severe and dangerous hæmorrhage from incisions of this kind. In a case which had come under his care at the Middlesex Hospital, he had excised a portion of the os uteri, leaving the part above the division healthy. He had occasion in this case to apply the actual cautery three times, the blood pumping out to a fearful extent from an artery as large as the radial. The application gave no pain. Four or five days after, hæmorrhage again came on, but was checked by the injection of cold water. It returned, however, in a few hours, and it was only arrested by plugging the vagina.

Dr. Locock should have said, if he had not said it already, that there was no hæmorrhage in these cases which was not controllable by plugging the vagina. In the case related by Mr. Arnott, this proceeding should have been adopted in the first instance.—*London Lancet.*

